# Implementing a Care Pathway for Complex Chronic Patients from a Nursing Perspective: A Qualitative Study

**DOI:** 10.3390/ijerph18126324

**Published:** 2021-06-11

**Authors:** Rosario Fernández-Peña, Carmen Ortego-Maté, Francisco José Amo-Setién, Tamara Silió-García, Antoni Casasempere-Satorres, Carmen Sarabia-Cobo

**Affiliations:** 1 Faculty of Nursing, University of Cantabria, 39008 Santander, Spain; carmen.ortego@unican.es (C.O.-M.); franciscojose.amo@unican.es (F.J.A.-S.); tamara.silio@unican.es (T.S.-G.); carmen.sarabia@unican.es (C.S.-C.); 2 IDIVAL Nursing Research Group, 39011 Santander, Spain; 3 SALBIS Research Group, University of León, 24400 León, Spain; 4 CualSoft-Qualitative Research Consulting Services, 03803 Alcoy, Spain; antoni@cualsoft.com

**Keywords:** care pathway, integrated health care, long-term care, quality of health care, Spain, qualitative research

## Abstract

A care pathway constitutes a complex care strategy for decision-making and the organization of processes in the care of complex chronic patients, avoiding the fragmentation of care. Health professionals play a decisive role in the implementation, development, and evaluation of care pathways. This study sought to explore nurses’ opinions on the care pathway for complex chronic patients three years after its implementation. The study participants were thirteen nurses with different roles who were involved in the care pathway. Thematic content analysis of the semi-structured interviews resulted in four major themes: (a) the strengths of the route; (b) the impact of the route on caregivers; (c) the weaknesses of the route; and (d) the future of the route. Overall, the pathway was positively valued for the benefits it provides to patients, the caregiver, and the administration of professional health care. Participants voiced their concerns regarding: communication and coordination difficulties among professionals across the different levels of care, the need for improved teamwork and consensus among professionals at the same center, and human and material resources. The ongoing evaluation and monitoring of facilitators and barriers is necessary throughout the implementation process, to ensure continuity and quality of care in the health system.

## 1. Introduction

Currently, our society is facing demographic, political, social, and economic changes that highlight the need to have health services that guarantee that the population’s health needs are met, especially concerning chronic processes. Progressive population ageing, together with the increasing prevalence of chronic diseases, are the main challenges faced by health systems across Europe [1].

Integrated care is ideally suited to respond to care needs in this context via proactive and well-coordinated patient-centered multidisciplinary care, using new technologies to support patient self-management and to improve the collaboration between caregivers [1]. This transformation requires a multi-level strategy with the simultaneous and synergistic implementation of various interventions within the same territory and population [2].

Following global trends, in recent years, mortality and disability in Spain are related to NCDs (Non-Communicable Diseases), which, in 2016, according to the WHO (World Health Organization), represented up to 91% of total deaths [3]. This reality has led to a redirection in the organizational approach of the health system as well as the design of specific policies for the care of these diseases [4]. 

The European Pathway Association defines the care pathway as a complex intervention for the mutual decision-making and organization of care processes for a well-defined group of patients during a well-defined period, although a variety of terms have been used interchangeably in the international literature without a clear differentiation, such as critical pathway, clinical pathway, integrated care pathway, or care map [5,6]. Different studies have highlighted the strengths of implementing the care pathway, revealing an overall positive impact on the organization, coordination, and monitoring of care processes, the involvement of professionals and the improvement of process indicators [7,8,9,10,11,12,13]. Health professionals play a decisive role in the implementation, development, and evaluation of chronic care strategies [14,15]. Specifically, nurses carry out an important task, both as coordinators for patient care and as care liaisons between health levels, supported by their own experience in the care path and managing patients with complex chronic needs [16,17,18].

Adopting the Chronic Care Model as a framework for the reorientation of the health care system [15,19], chronicity management models in Spain have been developed via plans or strategies at a national and regional level in different waves from 2006 to 2015. Most of these plans are based on population models focused on the analysis of the needs of people with chronic diseases, according to the risk stratification based on the Kaiser Permanente Pyramid [20] and the model of the British Kings Fund [21]. The Strategy for Addressing Chronicity of the Spanish National Health System [22] contemplates management by integrated care processes and the definition of care pathways for the different chronic health conditions that are included in most plans or strategies for the care of chronicity across different regions in Spain [21,23].

The autonomous community of Cantabria, located in the north of Spain, has a population of 584,308 inhabitants in 2020, with an aging rate of 22.5% (population aged 65 and over, compared to the total population) [24,25].

Currently, in the community of Cantabria, there are 5802 patients identified as pluripathological. Following the guidelines of the Chronicity Care Plan in Cantabria [26], in 2016, the “Chronic Patient Care Pathway” project was created, which was aimed at all chronic patients, seeking to address different organizational models for providing care to people with different chronic diseases. In the framework of this broader project, the Pluripathological or Complex Chronic Patient Care Pathway was implemented, designed for adult patients with two or more chronic pathologies and a high level of complexity. Its objectives were to modify the natural course of their diseases, delaying their progression and improving their health by maintaining patients in their usual environment. Three nursing roles are involved in the care of the complex chronic patient included in the pathway. Firstly, the hospital liaison nurse is present in the four community hospitals with the main role of ensuring continuity of care and coordination between the different levels of care. Secondly, due to their position of leadership among other health professionals, nurse managers are essential to strengthen the quality, coordination, and integration of care [27]. Lastly, community primary care nurses are considered well-positioned regarding the nursing care of patients included in the care pathways as well as for their insights regarding the evaluation of the care pathways [28].

Although the assessment of the chronic care strategy in Cantabria has been carried out in recent years from a quantitative approach, the assessment of the Complex Chronic Patient Care Pathway has not been studied from a qualitative approach, or, more concretely, from the point of view of nursing professionals.

The aim of this study was to explore nurses’ opinions on the care pathway for complex chronic patients in Cantabria, three years after its implementation, and on the different nursing roles involved in the complex chronic care pathway, specifically, the primary care nurse, the hospital liaison nurse and the nurse managers.

## 2. Materials and Methods

### 2.1. Design

A qualitative phenomenological descriptive design was conducted using semi-structured interviews. The analysis of the phenomena as they emerge from the point of view of nursing professionals’ experience is necessary for a deeper understanding of their meaning [29,30]. This study adhered to the Consolidated Criteria for Reporting on Qualitative Research (COREQ) guidelines developed to evaluate qualitative research reports [31].

### 2.2. Research Team

The research team was formed by PhD university teachers with a background in Nursing, Psychology (CO and CS), and Anthropology (RF). In addition, a sociologist, expert in qualitive data analysis and with a PhD in Education, participated in this research. All researchers had experience in research in health sciences and none were involved in professional activity associated with the participants.

This study was part of a larger project primarily aimed at studying the impact of nursing care in the complex chronic patient on dependency, perceived satisfaction, and caregiver burden in Cantabria and the Balearic Islands. The design of this mixed methods study included participants who were patients in the Complex Chronic Patient Care Pathway, together with their caregivers [32,33]. During the fieldwork phase and after the collection of the initial quantitative and qualitative data, the research team was involved in two work sessions, in which they raised and discussed the need to include the nursing professionals involved in the complex chronic patient pathway as participants in the project. In these sessions, the research team reflected on the beliefs and motivations for this study, considering that the inclusion of this type of participants would enable the possibility of gathering a broader and more profound view of nursing care in the care pathway for complex chronic patients.

### 2.3. Participants

The inclusion criteria consisted of nurses working in the Cantabrian Health Service as nurse practitioners with care and management experience in handling patients labeled as complex chronic patients and included in the route, and who occupied managerial positions in the implementation of the pathway. The exclusion criterion was nurses without professional experience in the care of these patients.

In total, 13 nursing professionals participated in this qualitative study, with different nursing roles: two nurse managers, four hospital liaison nurses and seven primary care nurses, none of whom withdrew from the study. Four primary care nurses were not included in the study because they lacked experience in the management of patients included in the complex chronic patient pathway.

Sample selection was performed using purposive sampling. Key informants from the Cantabrian Health Service who held management positions were contacted to participate in the study in this professional role. These participants provided us with access to primary care nurses. For the selection of the primary care nurses, the segmentation criteria included nurses belonging to the four health areas of the public health service. The snowball technique was used to access new primary care nurses working in the different health areas of the community. Finally, we contacted the four liaison nurses of the Cantabrian Health Service working in the public hospitals of the community. Participants were contacted by telephone at their workplaces inviting them to participate in the study and informing them of the study aims and procedures.

### 2.4. Ethical Considerations

This project was approved by the Clinical Research Ethics Committee of University of Cantabria (Internal Code 2017.049) and was authorized for its implementation in the Cantabrian Health Service. Prior to the study, the research aims and procedures were once again informed verbally and, in writing, and informed consent was obtained from each participant. The data were treated anonymously and confidentially, conforming to the Spanish legislation and the principles of the Declaration of Helsinki.

### 2.5. Data Collection

The semi-structured interviews were conducted following a script and field notes were gathered to record observations related to the data collection phase. Table 1 presents the topics and questions that were asked to the participants to obtain information based on their specific nursing role.

Data collection was carried out during the year 2019. The face-to-face interviews were conducted by two members of the research team (RF and CS) with experience in qualitative research and with the sole presence of the participant. The interviews were mostly conducted at the hospitals and primary care centers where the nursing professionals worked, and, in four cases, they were held on university premises. The interviews were conducted and analyzed in Spanish, lasted an average of 25 min, and were audio recorded and fully transcribed by two researchers from the research team. Finally, the interview extracts shown in this article were translated into English by expert translators. All information in the transcripts regarding the participants’ names and locations that could facilitate their identification was anonymized. Personal data were kept in a separate register accessible only to the project’s principal investigators and those involved in data collection (CO, CS, and RF). The interviews were stored and managed in MAXQDA software (VERBI Software—Consult—Sozialforschung GmbH, Berlin, Germany), and were only accessible to researchers involved in coding and analysis.

The field work was completed when data saturation was reached, meaning that the information provided in the interviews was already collected in the agreed upon analysis categories or in the emerging categories and did not provide new concepts for analysis [34].

### 2.6. Data Analysis

Once transcribed, the interviews were exported to qualitative software analysis MAXQDA 2020. An inductive thematic content analysis was carried out [35,36] by four researchers from the group with the aim of enabling the emergence of categories and subcategories. Although the thematic structure served to initially organize the findings, the emergence of ideas from the data was what shaped the study themes via the appropriate analytical categories. For the analysis of the qualitative content, several analytical cycles were carried out [37,38,39]. First, an initial substantive coding was developed close to the data, which was complemented by in-vivo coding to track metaphors in the participants’ discourse or significant terms. Subsequently, a process of categorization and recoding was performed which integrated the indicators into different categories or topics while reviewing and refining the coding system. The analysis was performed during the main data collection phase; however, it was considered mandatory to return to the field to perform more interviews to verify whether the categories raised were properly saturated. The analysis of the new data showed that no further relevant categories emerged from the data.

COREQ guidelines were followed [31], and different techniques were used to establish the trustworthiness of the data by reviewing issues concerning data credibility, transferability, dependability, and confirmability [40]. Researcher triangulation was achieved via team meetings that took place during the data collection and analysis phases, and participant triangulation was ensured by including different nursing professional roles involved in the phenomenon under study. Additionally, during the data collection, participant validation was ensured by asking the nursing professional to confirm the data obtained. A thick description of the phenomenon under study was included, and the rationale for the study was described in the framework of the wider research project. In addition, regular quality checks were made to avoid researcher bias. Furthermore, the research team adopted a reflective attitude, together with systematic attention to the context of the construction of knowledge.

## 3. Results

This section describes the characteristics of participants and presents the main thematic findings of our study.

In total, 13 nursing professionals (12 women, 1 man) participated in this qualitative study with a mean age of 45.9 years (range 37–54 years), and with a mean of 20.5 years professional experience as a nurse (range 12–29 years) (see Table 2). At the time of the study, all participants were working in different positions within the chronic patient care strategy of the Cantabrian Health Service: the four hospital liaison nurses in the community, primary care nurses from the different health areas, and two nurse managers.

Thematic content analysis of the interviews resulted in four major themes: (a) the strengths of the route; (b) the impact of the route on caregivers; (c) the weaknesses of the route; and (d) the future of the route. The main categories and subcategories are presented in Table 3.

### 3.1. Strengths of the Route

An emerging idea in this category that was highly valued by nursing professionals was related to the benefits of the route for patients and their families. Among the objectives that were positively evaluated as strengths, the professionals highlighted that the care pathway avoids or reduces hospital admissions, the number of visits to the hospital emergency department, as well as the number of days spent in hospital. As a result, patients spend more time in their usual social and family environment, leading to a substantial improvement in their quality of life. In addition, it favors a more individualized health care, suited to the patients’ needs, and prevents them from becoming unstable.

*The route increases the quality of life of patients within their pathological process (...) We are able to manage all resources better. We avoid unnecessary admissions, and therefore, at the hospital level there will be fewer admissions, which means less nosocomial diseases derived from the admissions. The greatest beneficiary is the patient, after that I think it is the family, and society. Regarding admissions, I believe there is good management, of course, it is more efficient to keep patients who are at home always well cared for, to avoid unnecessary admissions*.

(Informant 10)

*The route is a tool that makes it possible to organize the path of these chronic patients throughout their disease process. We try to establish a connection between primary and specialized care, because what we want is for these patients to remain in their environment as long as possible, because that is what will give them stability*.

(Informant 7)

Secondly, participants stressed that the route is a strategy that significantly improves the professional care provided to patients and also adds satisfaction in their work as professionals. Once patients are labeled as being pluripathological, this helps alert nurses to the need to provide health care adapted to the patients’ needs. Thus, patients are kept more stable, by providing care that is individualized and appropriate for their needs, without having to enter the usual care path. Moreover, the route facilitates the continuity of health care between primary care and the hospital.

*Well, a care pathway, a guide, that serves us to refer patients and not to lose them in this maelstrom that we can call the health system. To have them under control in primary care and that when they get out of control of primary care, which we understand is where the patients are, and they have to go to hospital, well, they must do so without losing continuity of care and without disassociating themselves from primary care*.

(Informant 1)

*The pluripathological label is a very important sign of alarm that puts us nurses with patients at risk, something that previously you could ignore […]. Whereas now, you continue to take care of them and follow their progress*.

(Informant 8)

### 3.2. Impact of the Route on Caregivers

The emerging ideas in relation to caregivers are related to aspects of the route that translate into improved care performance. Firstly, the route speeds up the procedures and facilitates the patient’s transit between the different institutions of the health system, which avoids overloading the caregiver and represents a benefit in the care provided.

*Yes, I think it is very beneficial for the caregiver or family because when they go to the hospital they don’t have to wait so long and then they’re discharged because they’re already on their way to where they’re going: to the residence or home or they get help... or they go to the social worker. They connect them in another way, without the relative or caregiver being concerned about what they have to do, or where they have to go*.

(Informant 13)

Secondly, the route makes it easier for caregivers to have a more accessible and closer relationship with health professionals and social workers at both the primary care and hospital levels. Likewise, this closeness makes caregivers feel more supported, with care adapted to their availability, feeling more confident with the care they provide to the patient and assume a more active role in care:

*For the caregivers, I think they perceive important support. In the end, what they see is that they truly have an avenue of help, a resource that is close to primary care*.

(Informant 7)

*There is a very important part which is also the relevance given to the caregiver in this program. Of course, the improvement in the quality of life is evident. (...) the support that they have, the security that it gives them to have you as a reference. For example, in the hospital, they know that they can call you directly [...], it gives the caregivers security and saves them a lot of paperwork*.

(Informant 5)

### 3.3. Weaknesses of the Route

On this subject, the informants underlined the lack of relationship and coordination between primary care and hospital nursing professionals, as a weakness in the process of implementing the care pathway. The elements involved in this lack of relationship refer to an already traditional separation between these two levels of care, difficulties in making contact between the reference nurses in primary care and the liaison nurse in the same health area as well as the lack of common spaces for contact between nurses of both care levels.

*To me, it seems essential for a connection to exist between hospital and primary care nurses. A true connection, not only in times of instability to avoid an admission, and not just between primary care physicians and specialized internists*.

(Informant 7)

Another point that stood out was the idea of insufficient joint work and consensus on the criteria and decision-making regarding complex chronic patients included in the route between professionals of the same center, especially in primary care centers, in addition to individual differences in terms of their involvement in the route as professionals. Specifically, referring to the control and power of decision that medical professionals have in the process, both in the labelling of patients and in the access to patient information during hospital visits:

*All patient information reaches the doctors so when a patient has been discharged, my doctor has to let me know that he has been discharged, otherwise I don’t know*.

(Informant 1)

Thirdly, the participants highlighted a weakness concerning implementation of the route, specifically, some aspects related to human and material resources that make it difficult for the operation of the route to achieve its objectives. Thus, the high turnover of health personnel, both across centers and between levels of care, due to changes in their employment situation, interferes with continuity in knowledge and in managing the route as professionals in the same centers. Moreover, communication difficulties were emphasized between hospital and primary care professionals, as the systems used in these different centers were often different.

*The computer applications should make it easier: if I, as a nurse, have a patient who is admitted and discharged, I should receive a discharge notice and a continuity of care report*.

(Informant 7)

### 3.4. Future of the Route

The need for a change of model in the approach to chronicity, the benefits of the route for patients and caregivers, and the conviction of their advantages as nursing professionals, makes the participants value their future in a positive way:

*[…] it’s the trend in health care right now […].. It’s a patient-centered model. These programs must succeed either way*.

(Informant 5)

*We are convinced that this is really good*.

(Informant 4)

The success of the route requires the design and implementation of certain areas of improvement. The need to improve communication and coordination between primary care nurses and hospital nurses was emphasized, by using improved resources for telematic communication, increasing the provision of nurses involved in the route, especially at the hospital level, as well as by providing continuous training on the route. Likewise, the need to redirect the role of nurses in the route by giving it more prominence and leadership was highlighted.

## 4. Discussion

The qualitative results of our study show that, overall, nurses have a positive assessment of the care pathway for both patients and caregivers. However, three years after its implementation, nurses stress the need for strategies to further improve in certain areas.

These areas include the need for more fluid communication and coordination channels and mechanisms between primary care nurses and hospital liaison nurses, teamwork, and consensus among professionals of the same center, as well as the improvement of human and material resources involved in the route.

The collaboration between clinical professionals from two different care levels has been carried out in Cantabria in recent years through the d’Amour questionnaire [41]. According to our findings, the quantitative results obtained from the questionnaire showed a mean score of 2.51 out of 5 in the years 2016–2019, thus suggesting equally improvable aspects in this area.

In this line, the care pathway should be considered as a process that represents a path of both success and challenges for professionals and managers, as shown in previous studies [42]. Given their dynamic nature over time, these new strategies essentially require periodic review and evaluation of their impact and assessment by both patients and professionals [11], considering the significant changes that their implementation may entail in the organizational culture and the possible need for support mechanisms to ensure their implementation in practice [7].

Inter-professional collaboration is a key element in efforts to increase the effectiveness of current health services, especially in the face of complex health problems, for which there are different conceptualizations, theoretical models [43] and assessment tools [41]. Regarding the care pathway of the complex chronic patient, the inter-professional collaboration between primary care and hospital care constitutes the vertebral axis upon which the comprehensive care between the health providers is based, thus avoiding fragmentation of care [44,45].

Other studies have focused on the role of the relational network between health professionals or health organizations from a structural point of view, in health settings and for the implementation of programs [46,47,48,49,50]. Thus, the multilevel model proposed by Van Houdt et al. [51] highlights the need for improved communication and coordination in the implementation of a care pathway between primary and hospital care professionals. These changes include improvements at the level of inter-organizational networks through the exchange of information and development of new communication channels, shared objectives, knowledge of the roles and competencies of the different professionals, and improved relationships. Secondly, changes in the inter-organizational mechanisms are required to change the structures of the organizations before and after the implementation of the care pathway in addition to the development of facilitating strategies to favor the creation of interpersonal networks between professionals, together with the transfer of information by electronic means [50].

The design and implementation of a new approach to health service delivery represents a challenge for managers and professionals, beginning with the preliminary planning of the implementation process, by considering the contextual, organizational, and cultural characteristics of the organizations and the intricacies of the care provided [42,52].

## 5. Conclusions

The care pathway of the complex chronic patient in today’s healthcare reality constitutes a valuable care strategy which responds to the needs of these patients and their caregivers in complex settings, by avoiding fragmentation of care. Due to their leadership roles, nurse managers are in an ideal position to ensure the correct implementation of these care pathways. A permanent evaluation and monitoring of facilitators and barriers to the implementation process is necessary to guarantee the continuity and quality of care in the health system.

## Figures and Tables

**Table 1:** Semi-structured interview guide.

Topic	Questions
Professional background	Tell us about your experience and professional role regarding the complex chronic patient
Impact of the pathway	What is your opinion on the impact of the care pathway in the patients and their caregivers?
Strengths and weaknesses	Tell us about the strengths and weakness in the functioning of the pathway
Improvement strategies	Could you identify relevant strategies for improving the care pathway?
Future of the pathway	How do you rate the future of the pathway for the complex chronic patient?

**Table 2:** Profile of informants.

Informant	Sex	Age	Occupation	Professional Experience (Years)
Inf. 1	F	46	Primary Care Nurse	26
Inf. 2	F	37	Hospital liaison nurse	16
Inf. 3	F	40	Hospital liaison nurse	17
Inf. 4	M	49	Nurse manager	12
Inf. 5	F	48	Hospital liaison nurse	26
Inf. 6	F	54	Primary Care Nurse	21
Inf. 7	F	40	Nurse manager	20
Inf. 8	F	48	Primary Care Nurse	20
Inf. 9	F	46	Hospital liaison nurse	22
Inf. 10	F	45	Primary Care Nurse	17
Inf 11	F	48	Primary Care Nurse	29
Inf. 12	F	49	Primary Care Nurse	20
Inf. 13	F	47	Primary Care Nurse	21

M: Male, F: Female.

**Table 3:** Distribution of the main categories and subcategories.

	Categories
Strengths of the route	Benefits for the patient and family Benefits in the development of professional health care
Impact of the route on caregivers	It expedites the procedures and facilitates the patient’s transit through the health system More accessible relationship with professionals and improved care
Weaknesses of the route	Insufficient coordination between primary care and hospital nursing professionals Need for improvement in teamwork and consensus among professionals in the same center Need for improvement of human and material resources
Future of the route	Positive future of the route Need for improvement strategies

## Data Availability

The data presented in this study are available on request from the corresponding author. The data are not publicly available due to privacy and ethical considerations.
